# Reconstructing Image Composition: Computation of Leading Lines

**DOI:** 10.3390/jimaging10010005

**Published:** 2023-12-25

**Authors:** Jing Zhang, Rémi Synave, Samuel Delepoulle, Rémi Cozot

**Affiliations:** Laboratoire d’Informatique Signal et Image de la Côte d’Opale (LISIC), Université du Littoral Côte d’Opale, UR 4491, F-62228 Calais, France; jing.zhang@univ-littoral.fr (J.Z.); remi.synave@univ-littoral.fr (R.S.); samuel.delepoulle@univ-littoral.fr (S.D.)

**Keywords:** image composition, leading lines, grouping lines, image aesthetic

## Abstract

The composition of an image is a critical element chosen by the author to construct an image that conveys a narrative and related emotions. Other key elements include framing, lighting, and colors. Assessing classical and simple composition rules in an image, such as the well-known “rule of thirds”, has proven effective in evaluating the aesthetic quality of an image. It is widely acknowledged that composition is emphasized by the presence of leading lines. While these leading lines may not be explicitly visible in the image, they connect key points within the image and can also serve as boundaries between different areas of the image. For instance, the boundary between the sky and the ground can be considered a leading line in the image. Making the image’s composition explicit through a set of leading lines is valuable when analyzing an image or assisting in photography. To the best of our knowledge, no computational method has been proposed to trace image leading lines. We conducted user studies to assess the agreement among image experts when requesting them to draw leading lines on images. According to these studies, which demonstrate that experts concur in identifying leading lines, this paper introduces a fully automatic computational method for recovering the leading lines that underlie the image’s composition. Our method consists of two steps: firstly, based on feature detection, potential weighted leading lines are established; secondly, these weighted leading lines are grouped to generate the leading lines of the image. We evaluate our method through both subjective and objective studies, and we propose an objective metric to compare two sets of leading lines.

## 1. Introduction

**Figure 1:**
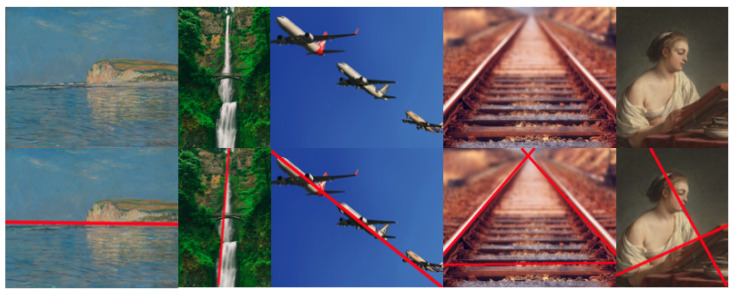
Leading lines: Our method automatically computes the likely leading lines that underlie the image’s composition. Depending on the complexity of the image’s composition, our method can reconstruct either a single leading line or multiple leading lines. The top is the original image and the bottom is the leading line result.

Images serve as a means of storytelling. Martine Joly et al. [1] propose that, apart from the depicted scene and its staging, various aesthetic and artistic elements aid creators in conveying their intended message. These elements encompass aspects such as the color palette, lighting mood, framing, point of view, and composition. Consequently, the reconstruction of these aesthetic components can facilitate image analysis and comprehension. The reconstruction of specific aesthetic features has been the subject of numerous publications, including the computation of a color palette [2,3], which describes the primary colors in an image, and the determination of the lighting style [4] associated with an image’s aesthetic. Concerning models of image composition, the primary challenge arises from the diverse approaches used to describe image composition. Some authors employ shape-based models [5], describing composition in terms of primary shapes or their juxtaposition, while others utilize line-based models [6]. Shape-based image composition models also incorporate leading lines to elucidate the arrangement of shapes (see Figure 1), providing an aesthetic rationale for these leading lines. For instance, Molly Bang [5] expounds that an underlying upward diagonal leading line implies motion or tension.

First research on image aesthetics evaluation employing hand-designed features underscored the pivotal role of composition in assessing aesthetics. In these studies, authors do not attempt to reconstruct the composition’s leading lines but instead rely on classical composition rules, such as the rule of thirds or upward and downward diagonals [7]. They assess the alignment of main objects along vertical and horizontal lines that divide the image into thirds [8,9]. While the detection of leading lines is crucial for image content understanding and aesthetic evaluation, there is currently no dedicated method for recovering these lines. Debnath et al. [10] acknowledge the impact of leading lines on aesthetic scores and propose a convolutional neural network for leading line recognition. However, their method merely estimates the existence of evident leading lines in an image without pinpointing their precise locations. The rule of thirds is also widely utilized in automated image cropping [11,12].

**Figure 2:**
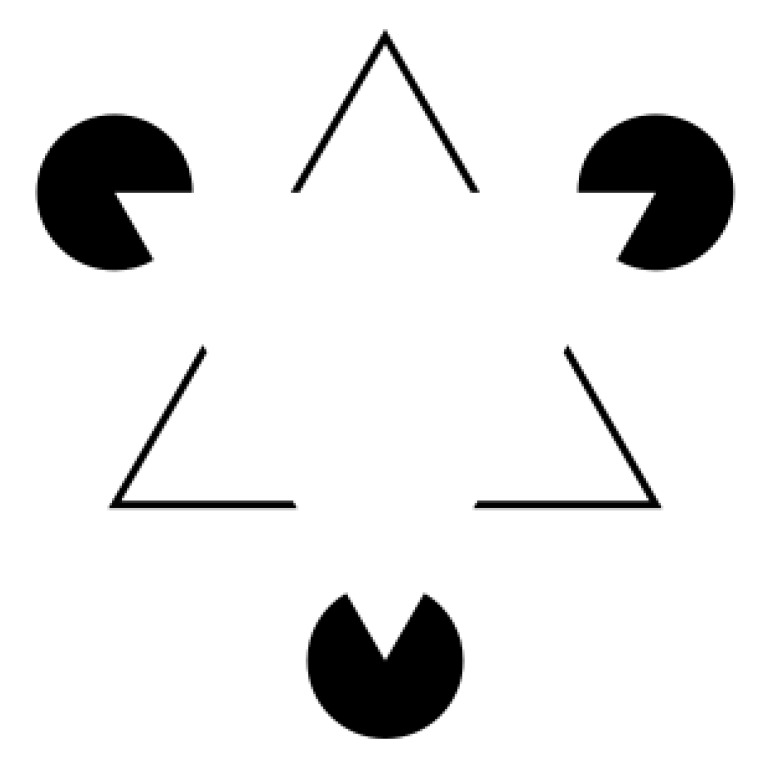
The Kanizsa figure is an optical illusion that demonstrates how our brains perceive invisible lines. Even though there are no actual lines connecting certain shapes, our brains create the perception of hidden lines due to our innate ability to fill in gaps and interpret incomplete information.

The role of lines in the human visual system is fundamental for perceiving and interpreting the surrounding world. Lines are essential visual elements that the brain utilizes to construct shapes, objects, and scenes. This process of visual perception and organization aligns with Gestalt psychology, which provides historical context for our comprehension of how the brain processes visual information [13]. The importance of lines in perception is evident in the concept of “Illusory contours”, where edges, lines, or shapes appear to exist in a visual scene even when they are not physically present in the stimulus. In other words, our visual system fills in missing information to create the illusion of contours or boundaries that are not physically present. One of the most well-known examples is the Kanizsa figure or “Pac-Man configuration” (see Figure 2). Recent research has indicated that certain neuroanatomical structures are specialized for detecting lines. Line perception involves both lower-level processing in the primary visual cortex (V1) and higher-level processing in areas within the visual association cortex [14].

**Figure 3:**
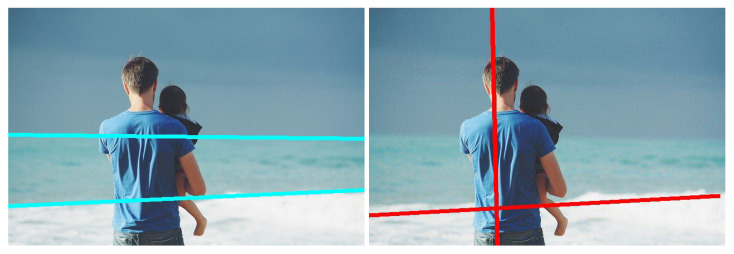
Comparison between semantic lines (depicted by blue lines in the left-hand images, computed according to [17]) and composition leading lines (illustrated by red lines in the right-hand images) computed with our method: while certain semantic lines may also serve as composition leading lines, they alone are insufficient for a comprehensive depiction of the image’s composition. Indeed, there are composition leading lines that do not align with semantic lines.

Semantic lines are closely related to leading lines, as they demarcate lines in an image that separate different semantic regions [15]. Numerous studies have focused on the detection of semantic lines [15,16,17,18]. While in some instances, semantic lines may also serve as leading lines for composition, the utilization of semantics to expound image composition has sometimes imposed the overly restrictive assumption that leading lines are confined to the borders of regions with different semantics. Leading lines, in fact, can connect different points without necessarily denoting a boundary between zones. It is important to note that, in most cases, a subset of semantic lines can also be considered leading lines. However, leading lines in an image encompass lines that are not categorized as semantic lines (see Figure 3).

To our best knowledge, no computational method has been introduced for tracing image leading lines. Furthermore, there is no predefined ground truth for the leading lines of image composition, as these are features derived from image analysis [1]. The consensus among image experts in defining leading lines for composition is a valid question, as individual interpretations may vary. Subjective studies show substantial agreement among experts on most images, supporting the feasibility of automated leading line detection.

In this paper, we present a method that automatically identifies the likely leading lines in an image’s composition (see Figure 1). Our method encompasses several steps: (1) the computation of the contrast map of the image; (2) the generation of potential leading lines weighted according to the contrast map and (3) the grouping of potential leading lines to extract the final leading lines.

Our main contributions are the following.

First, we show that there is a strong consensus among experts on where the composition leading lines of an image are. Consequently, this confirms that modeling image composition by a set of leading lines is a worthwhile approach.

Second, we propose a dataset of forty images and related composition leading lines drawn by four experts. The dataset includes photographs, paintings, and drawings.

Third, we propose the first non-supervised method to compute the likely composition leading lines of an image. We designed the method as simple as possible to have an understandable method.

In Section 2, we provide a brief overview of existing methods designed to detect lines in images. The primary focus of these methods is the detection of real edges in images rather than underlying lines. In this section, we also summarize the approaches related to semantic line detection. In Section 3, we introduce a metric for measuring the distance between two sets of lines and assess the consensus among experts in identifying composition leading lines within images. The results of this study underscore the validity of proposing an automatic algorithm for reconstructing composition leading lines. Section 4 explains the method used to compute the composition leading lines, while Section 5 delves into the analysis of the results. Furthermore, we demonstrate that this algorithm can be a valuable tool for guiding image capture in Section 6. Finally, Section 7 concludes this work and sets the stage for future works.

## 2. Related Works

Numerous methods have been proposed for the detection of lines in digital images. The Hough Transform [19] stands as one of the most commonly employed algorithms for line detection. It transforms the task of detecting straight lines in image space into the challenge of detecting points within a parameter space. However, the Hough transformation can be time-consuming. To mitigate this issue, the probabilistic Hough Transform was introduced [20]. Other innovative approaches, such as those presented in [21,22], apply an elliptic-Gaussian kernel and a pyramid structure to enhance the original Hough transform. In line detection through the Hough Transform, the typical initial step involves edge detection. Apart from Hough Transform-based line detectors, several approaches, such as [23,24,25], have proposed mathematics-based line segment detectors.

Furthermore, there are learning-based approaches, such as those found in [26,27,28,29], which leverage convolutional neural networks (CNNs) to predict line segments within images. For example, [26] utilizes a U-net architecture to predict segment masks and tangent fields, subsequently applying a grouping algorithm to convert them into the final line segments. On the other hand, [27] introduces a representation of line segments using center, angle, and length, combined with a shared feature architecture for line segment prediction. These methods’ objectives are to recover line segments (local features) in images to extract the wireframe structure of objects or to estimate human poses [30,31]. Composition leading lines do not necessarily consist of objects’ edges so we will not rely on these approaches. Edges of objects or boundaries of large-scale semantics zones are meaningful curves in images that structure the images. Computing these geometric structures in a digital image without any a priori information led to many publications [32,33]. computed maximal boundaries using local contrast and the Helmholtz Principle. The large-scale structure given by these methods is not the set of composition leading lines but is close to a sketch outlining the boundaries of image objects.

Semantic lines, defined as the primary and significant lines that demarcate various semantic regions within an image [15], have also received considerable attention. The work of [15] marked the inception of semantic line detection, with the introduction of a multi-task learning CNN for predicting semantically important lines. To facilitate network training, they assembled a semantic line dataset comprising 1750 images and employed the mean intersection over union (mIoU) metric for line distance measurement. Building upon the work of [15,17,18], devised a harmonization network and a complete graph for determining the final semantic lines. They also introduced a harmony-based intersection-over-union (HIoU) metric to gauge the overall matching score of two sets of lines. Additionally, [16] incorporated Hough Transform into a deep learning network for predicting semantic lines in the parametric space, thereby transforming line detection into point detection. They curated a dataset containing 6500 images across various scenes and proposed a distance metric that considers both Euclidean and angular distances for measuring line distances. A common thread among these works is their adoption of supervised learning approaches, which necessitate the availability of suitable datasets.

However, these traditional line detection methods are unable to reconstruct the leading lines in the image. Straight line detections focus on the actual physical lines in the image, edge detections focus on portraying the edge details of the object rather than a line that has leading effects. Semantic lines are approximated to leading lines in special scenarios but are incompletely equivalent. Semantic line detections emphasize detecting lines that segment semantic regions of an image rather than analyzing image compositional features. Therefore, we propose a method aimed at reconstructing the image leading lines.

## 3. Leading Lines to Model Image Composition

We propose to model image composition, encompassing various visual mediums such as painting, engraving, drawing, and photography, using the concept of leading lines. In this paper, the comprehensive characterization of a line encompasses an infinite arrangement of points, yet its manifestation on a 2D image materializes as a collection of pixels originating from the image’s perimeter.

Composition-leading lines structure the entire image, they do not describe the structure of a part of the image, consequently, a composition-leading line goes through the image: starting on an image edge and ending on another image edge. Composition-leading lines structure the content inside the image while the edges define the frame, consequently, we assume that image edges are not composition-leading lines. Assessing the viability of using leading lines to model image composition involves examining expert consensus in defining the leading line when recovering image composition. Without author-provided ground truth, identifying leading lines becomes subjective, relying on viewer interpretation.

We show that expert agreement justifies automated leading line detection. Indeed, we conducted a subjective experiment where image experts outlined leading lines based on their composition interpretation and compared their similarities. For this purpose, we adapted the metric introduced by Zhao et al. [16] in Section 3.1.

### 3.1. Distance between Two Sets of Lines

In their work [16], Zhao et al. introduced a similarity score known as the EA-score to quantify the similarity between two lines. This score is defined as:

(1) $$S_{EA}\left(l_{i},l_{j}\right)={\left(\left(1-\frac{\theta (l_{i},l_{j})}{\pi /2}\right)\times \left(1-D(l_{i},l_{j})\right)\right)}^{2}$$

Here, $l_{i}$ and $l_{j}$ denote two lines, and $\theta (l_{i},l_{j})$ stands for the angle between these two lines, $D(l_{i},l_{j})$ represents the Euclidean distance between the midpoints of two lines (both lines are cropped to fit the image frame). It is essential to note that the image is first normalized as a unit square before the computation.

Now, our objective is to assess the agreement between sets of manually defined composition leading lines. As a result, we require a measure to evaluate the similarity between two sets of lines. Leveraging the similarity score $S_{EA}$, our initial step is to define the distance $d_{LS}$ between a line $l_{i}$ and a set G comprising *N* lines. We adopt the classical method of computing the distance between one element and a set of elements based on the distance between two individual elements. This is expressed as:

(2) $$d_{LS}\left(l_{i},G\right)=\min_{n\in \{1..N\}}\left(d_{EA}\left(l_{i},g_{n}\right)\right)$$

Here, $d_{EA}\left(l_{i},l_{j}\right)=1-S_{EA}\left(l_{i},l_{j}\right)$ and $g_{n}$ denotes a line belonging to the set G.

Subsequently, we define the distance between two sets of leading lines, $D_{LS}\left(F,G\right)$, as the average of two average distances. The first average distance computes average of $d_{LS}\left(f_{i},G\right),i=0,1,\ldots ,N_{F}$, whereas the second average distance computes average of $d_{LS}\left(f_{i},F\right),i=0,1,\ldots ,N_{G}$, the equation to express this distance is as follows:

(3) $$D_{LS}\left(F,G\right)=\frac{1}{2}\left(\left(\frac{1}{N_{F}}\sum_{i=1}^{N_{F}}d_{LS}\left(f_{i},G\right)\right)+\left(\frac{1}{N_{G}}\sum_{i=1}^{N_{G}}d_{LS}\left(g_{i},F\right)\right)\right)$$

In the equations above, F and G represent sets of $N_{F}$ and $N_{G}$ composition leading lines, respectively. This formulation ensures that we obtain a symmetric distance measure, meaning that $D_{LS}\left(F,G\right)$ is equivalent to $D_{LS}\left(G,F\right)$. This is consistent with the similarity comparison logic, when comparing the results of leading lines in two images, the order of the images does not affect the comparison result.

### 3.2. Preliminary Study about Experts Agreements

Our preliminary study aimed to evaluate consensus among experts in image creation and analysis regarding composition leading lines, considering the subjective nature of their perception. In the experiment, four experts were tasked with delineating leading lines on a diverse set of forty images. Using the metric described in Section 3.1, we quantitatively assessed agreement among the experts, who had no time constraints in defining the lines.

**Figure 4:**
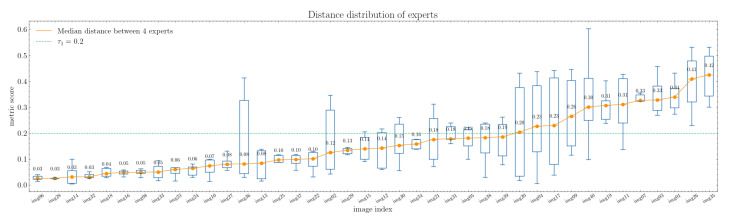
Analysis of the distances between composition leading line sets traced by experts. The images on the x-axis are arranged in order of the median distance. Each image is represented by a box plot displaying the distribution of distance scores among the experts. The orange line within each box plot represents the median distances between the composition leading lines identified by the four experts.

**Figure 5:**
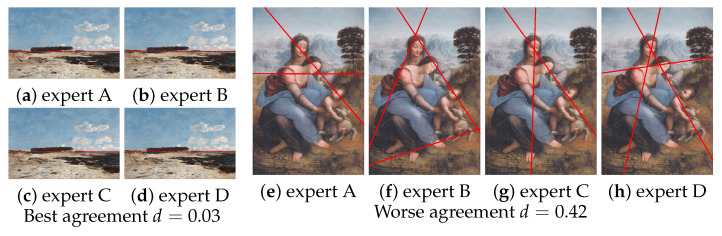
Projection of the leading lines determined by each expert, to visualize the agreement between them. Best agreement among experts: a to d images show the leading lines defined by each expert. Each expert has defined a single leading line which is confused with the horizon, the median distance between four sets of leading lines is 0.03. Worse agreement among experts e to h images show the leading lines defined by each expert. In this case, two experts defined two leading lines while the two others defined 3 leading lines, the median distance between four sets of leading lines is 0.42, nevertheless, we can note that all experts traced a downward diagonal as a leading line.

The median distance between composition leading lines delineated by experts exhibits an overall low value, signifying a high degree of consensus among them. The distances vary within the range of 0.03 to 0.42, as illustrated in Figure 4. However, on the right-hand side of the figure, we observe a more pronounced disparity between experts. This discrepancy is particularly noticeable in certain images with intricate compositions, where even the assessments of experts may differ significantly (see Figure 5).

Except for eleven images, the inter-expert distances fall below or equal to 0.2. For half of the images, the distances are less than 0.14. The median distance exceeds 0.4 for only two images (images 26 and 35). This suggests a substantial level of agreement among the experts.

The findings of this study indicate a strong consensus among the experts when identifying composition leading lines in images. The substantial concurrence in their assessments highlights the reliability and consistency of their judgments. Given the high agreement among these experts, it is justified to put forth a model for detecting composition leading lines.

In summary, the results of this preliminary study support the notion that experts exhibit a strong consensus in their identification of composition leading lines in images. This paves the way for developing a model in this domain.

## 4. Method: Computing the Composition-Leading Lines

**Figure 6:**
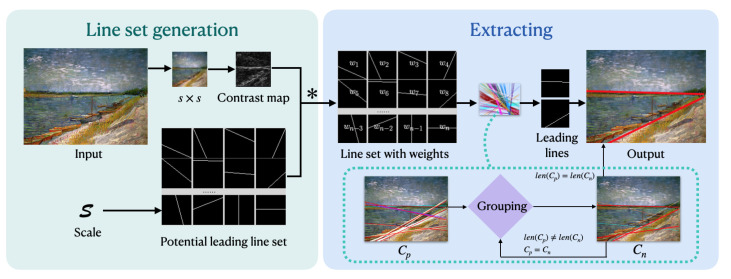
Overview of our method’s pipeline. The operator “*” between “line set generation” and “extracting” denotes the weight computation of a line (see Equation (5)).

In this section, we present our algorithm for detecting composition leading lines in images. Figure 6 provides an overview of our approach, which consists of two main steps: first, weighting all the potential leading lines, and second, grouping the lines to identify the final leading lines.

The first step comprises three components:

1. We employ spline interpolation to resize the image from its original resolution to s×s. This resizing retains the general structure of the image.
2. We compute a contrast map of the resized image.
3. We assign weights to all potential lines based on the sum of contrast values for each crossed pixel.

In the second step, we have developed a grouping algorithm to determine the final leading lines from the set of potential lines. Each iteration involves grouping closely positioned potential leading lines to form groups. The central leading line of each group becomes one of the final leading lines.

### 4.1. Generation of the Potential Line Set

**Figure 7:**
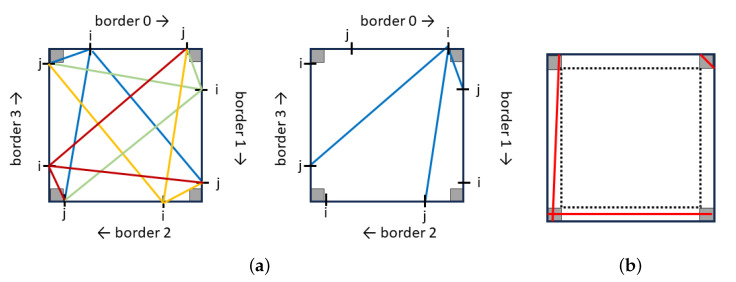
Generation of all possible leading lines. (**a**) We generate 12 leading lines from each couple (i,j), where *i* is the coordinate of the starting point on border *k* and *j* the ending coordinate on border l≠k. The couple (i,j) is in s×s. Note, that the couple (j,i) produces the same set of lines. (**b**) Lines near the edges, shown in red, are not considered potential leading lines.

We begin by generating all possible leading lines in the image. A leading line starts from one image edge and ends at another edge. Therefore, the total number of potential leading lines is given by: $s^{2}\times \frac{4\times 3}{2}=s^{2}\times 6$. For each couple (i,j)∈s×s, it produces 3 lines starting from pixel *i* along border $b_{n}$ and ending at pixel *j* along border $b_{m}$, with *n* and *m* in {0,1,2,3} and n≠m. Since there are four possible starting borders, each couple (i,j) yields 12 lines. It is important to note that the couple (j,i) produces the same lines as (j,i) (See Figure 7). To enhance the robustness of our results, we exclude very short lines and those that are in close proximity to the image edges, as illustrated in Figure 7. This reduces the search space to ${\left(s-\delta \right)}^{2}\times 6$. A typical value for δ is s/10.

### 4.2. Contrast Map: Discrete Derivative of Gradient $L_{1}$ Norm

After generating each potential leading line, we calculate a weight for each line. A higher weight signifies greater visual significance, indicating a higher likelihood of being a leading line. Various forms of pixel contrast values are utilized for detecting salient pixels in an image [34,35,36]. We propose computing the contrast map $M_{i,j}$ as follows:

(4) $$\begin{array}{cc} M_{i,j} & =|P_{i,j+1}-P_{i,j}\left|+\right|P_{i+1,j-1}-P_{i,j}|\\ \; & +|P_{i+1,j}-P_{i,j}\left|+\right|P_{i+1,j+1}-P_{i,j}| \end{array}$$

Here, $P_{i,j}$ represents the pixel *Y* value of row *i* and column *j* in the XYZ color space. Our contrast value closely resembles both the discrete derivative of the L1 norm of the gradient and the discrete Laplacian of the image.

Subsequently, the weight of a line is calculated as the cumulative value of the element-wise of the pixel contrast values.

(5) $$\begin{array}{c} W_{i}=\sum_{p\in l_{i}}M_{p} \end{array}$$

Here, *p* represents all the pixels in line $l_{i}$, and $M_{p}$ is the contrast value of pixel *p*.

We do not normalize line weight based on its length to prioritize longer lines. In fact, we operate under the assumption that composition lines encompass the entire image globally.

### 4.3. Extracting of Leading Lines

Having obtained all the weighted potential leading lines, we proceed with an extracting process (see Algorithm 1) to identify the final leading lines. The final leading lines are represented by the central line of each group. Our extracting algorithm operates iteratively. In each iteration, a new set of groups is generated, with a group count equal to or less than that of the previous iteration. The process terminates when the number of groups remains unchanged between two consecutive iterations. Each iteration follows these steps:

- We begin an iteration with a set of line groups: $C=\{C^{i}\}$ with $C^{i}=\left(l^{i},W^{i},\left[l_{j}^{i}\right]\right)$. The superscript *i* is the index of the group, and the subscript *j* is the index of the line. Each group is defined by:
  - its central line $l^{i}$ that is the line in the group whose weight is the median weight of lines $l_{j}^{i}$ belonging to the group.
  - the lines $l_{j}^{i}$ belonging to the group, also noted: $l_{j}\in C^{i}$
  - a weight $W^{i}$ equal to the maximum weight of leading lines weights in the group: $W^{i}=\max_{l_{k}\in C_{i}}W_{k}$
- We build a new set of lines $L=\{l_{p}\}$ consisting of the central lines of groups and related weight and start a new group set to an empty set: C={}
- For each line $l_{p}$, in decreasing order of weight $W_{p}$, we evaluate if $l_{p}$ belongs to an existing group $C^{q}$. The inclusion criteria for $l_{p}$ in $C^{q}$ is as follows: $\left(d_{EA}\left(l_{p},l^{q}\right)<\delta_{d}\right)\&(|W_{p}-W^{q}\left|<\delta_{W}\right)$. If the criteria are validated then the line $l_{p}$ is put in $C^{q}$, and we pass to the next line. If $l_{p}$ is in the scope of $C^{q}$, i.e., $\left(d_{EA}\left(l_{p},l^{q}\right)<\delta_{d}\right)$ but with $\left(|W_{p}-W^{q}|>\delta_{W}\right)$ then the line $l_{p}$ is discarded and we pass to next line. Finally, if $l_{p}$ has not been discarded and does not belong to any existing group, a new group $C^{k}=\left(l^{k}=l_{p},W^{k}=W_{p},\left[l_{p}\right]\right)$ is built.
- At the conclusion of the iteration, we update these and the thresholds. Updating the median weighted line as the central line of the group. The distance threshold is fine-tuned by adding 1/S, while the weight threshold is fine-tuned by adding 1.

**Algorithm 1:** Leading line grouping
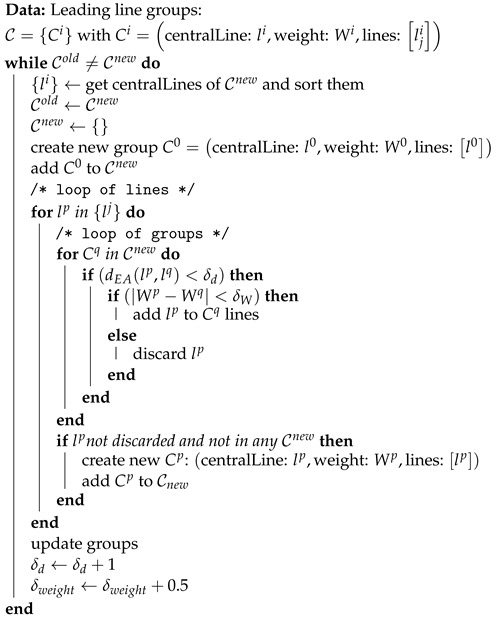


The initialization process of the algorithm is the following: (1) set the initial values for $\delta_{W}=3$ and $\delta_{d}=8/s$ where *s* is the size in pixel of the resized image, and (2) the initial set of lines are all the possible leading lines computed at the previous step. In practice, we limit the initial set of lines to the first two percent of lines from the previous step, in decreasing order, this speeds-up the algorithm and does not impact the final results. The algorithm ends when the new set of groups is equal to the previous iteration one.

In the next section, we present some results, specifically the composition leading lines computed by our algorithm, and then delve into a detailed analysis of these findings.

## 5. Results and Discussion

In this section, we first showcase the reconstructed leading lines from various types of images, including paintings and photography. Next, we assess our model’s performance through subjective studies. Finally, we compare our results with ground truth data.

### 5.1. Results

**Figure 8:**
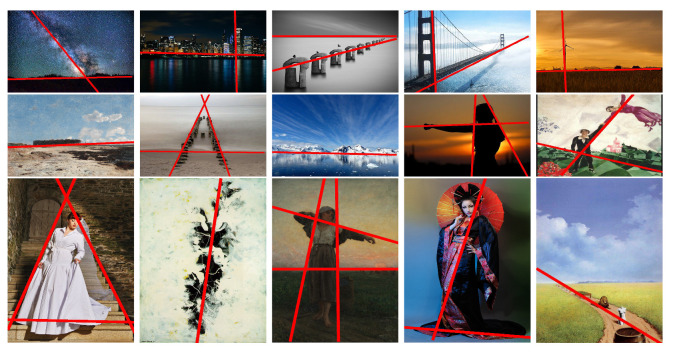
Results of leading lines: Test images include both paintings and photographs of various compositions, our method gives results that are visually consistent with human perception of leading lines.

Figure 8 displays a selection of results obtained using the following algorithm parameters: s=64 pixels, $\delta_{d}=8/s$, and $\delta_{W}=3$. Overall, the reconstructed composition leading lines are quite well aligned with our expectations. Our algorithm yields an average of 2.8 leading lines across a dataset of 40 images, ranging from a single leading line for straightforward compositions to up to 5 for more complex cases.

**Figure 9:**
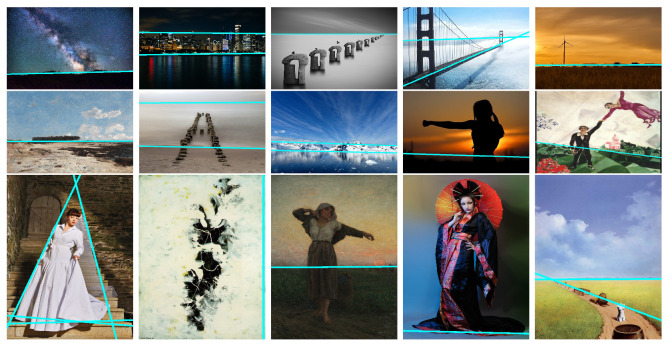
Semantic lines detection results [16].

Figure 9 showcases the semantic lines obtained from the same 15 images using Zhao et al.’s approach [16]. It is apparent that while in some instances, certain semantic lines align with leading lines, the semantic line approach often falls short in detecting composition leading lines. This observation underscores the need for our algorithm’s proposal to reconstruct composition leading lines.

### 5.2. Subjective Study

In this section, we measure how the model performs for naive observers. To do this, the leading lines predicted by the model are presented to a group of subjects with no specific knowledge. After a brief presentation of what constitutes a composition leading line, observers were asked to choose, through a 2AFC procedure (two alternative forced choices), the results of the model and another version.

To assess the model’s relevance for describing leading lines, a Two Alternative Forced Choice (2AFC) methodology is employed. The dataset includes 40 diverse images selected for their variety.

**Figure 10:**
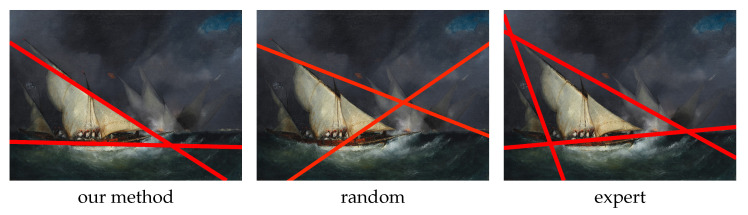
An example for the comparison.

For each image, three versions of the leading line are computed (See Figure 10): one with the method described above, the other with the same number of lines randomly arranged on the image and the last corresponds to the lines chosen by one of the experts. The version of the lines chosen to represent the experts is, for each image, the one that minimizes the distance from the other experts. This version is considered the most representative of expert opinion for the considered image.

The following comparisons are proposed:

- model versus random
- model versus expert version.

A total of fifteen volunteers took part in the experiment (11 males and 4 females). The average age is 23.4 years with a standard deviation of 11.1. All participants have normal vision or corrected-to-normal vision; four of twelve wear glasses.

**Figure 11:**
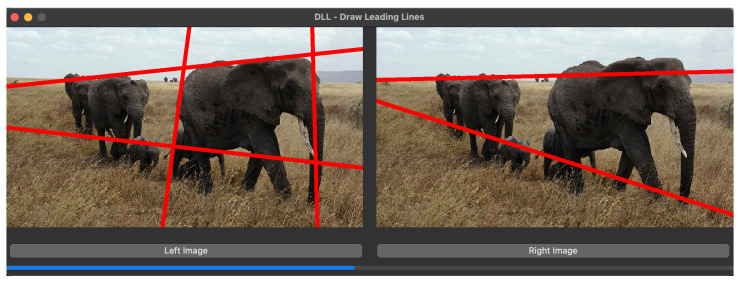
Interface for the experiment. Participants have to choose the image where the displayed line set corresponds as closely as possible to the image leading lines.

Images are shown on a standard laptop monitor (Full HD resolution). The subjects are given a short presentation of what the leading lines are with a few examples. They are then asked to choose the version they feel best represents the leading lines over the images, as shown in Figure 11. In order to avoid order and position bias, the order of presentation of the 40×2=80 images is randomized (order and side presentation).

**Figure 12:**
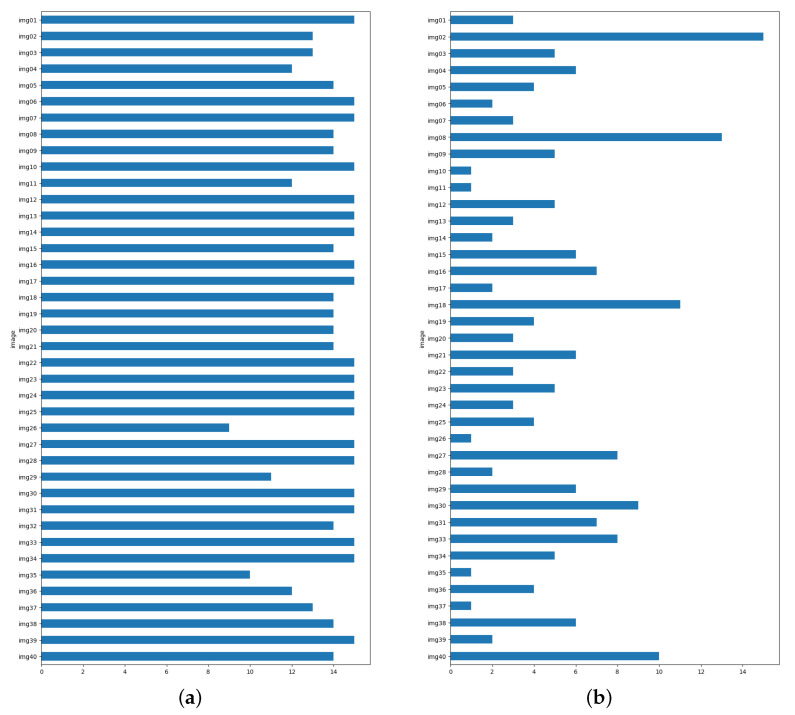
Results of the subjective study: (**a**) Choice for the model when compared with the a random distribution. (**b**) Choice for the model when compared with the most representative expert.

The results of the experiment are presented in Table 1 and Figure 12. Participants consistently preferred the lines suggested by our method when our method is confronted with a random distribution. On the other hand, when the choice is between our method and an expert’s proposal, the majority of subjects tend to choose the expert’s proposal, but our method is nevertheless chosen in around a third of cases. These two effects are statistically significant at a threshold of 1% error. ($\chi^{2}$ values are, respectively, 447.2 and 77.8).

A per image analysis shows that for all the images considered, the model is preferred to a random distribution. When the choice is between the lines of an expert and that of the model, a large variation is observed. Although the majority of the images are chosen by the experts, the model is preferred for certain images, in particular, images 2, 8, 18 and 40. Images 10, 11, 26, 35 and 37 remain problematic for the model.

The subjects’ choices are clearly in favor of the model when compared with a random distribution of the same number of leading lines. When compared to the most representative expert, the model’s choices are retained by the observers in about a third of the cases. This suggests that the algorithm’s choices are of good quality, although they do not match the accuracy of the experts. In some images, the model even seems to give an “opinion” that the experts had not considered.

### 5.3. Comparison of the Model with Ground Truth

**Figure 13:**
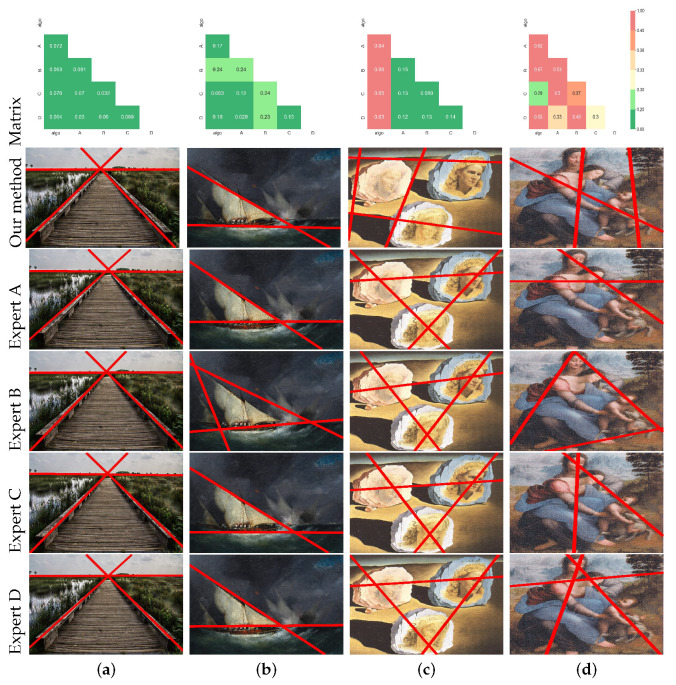
Comparison results between different sets of lines: The first row shows the distances between the different leading lines set, green values representing distances less than 0.3 and two line sets with consistent spatial distribution, and non-green values representing distances greater than 0.3 and two line sets with inconsistent spatial distributions. (**a**) indicate that the algorithm results are consistent with the expert’s results. (**b**) indicates that the results of expert B have subjective differences from the other results but are consistent overall. (**c**) shows agreement among the four experts, but the results of our algorithm are inconsistent with the experts. (**d**) shows that there are large subjective differences in this image, even among experts.

In Section 3.2, we established the consistency of subjective markings between four experts. Consequently, we utilized the results from the four experts as a reference to validate the consistency of our algorithm’s output with the manual results. We introduced our algorithm results as the fifth set of expert markers and employed our line set metric to compute the distances between these five sets. These distance values constituted a symmetric 5×5 matrix, with zeros along the diagonals. Observing this matrix, we noted that when the distance between two sets was less than 0.2, as depicted in Figure 13a, there were no significant visual disparities between them. When the distance ranged between 0.2 and 0.3, as illustrated in Figure 13b, their layouts were similar, albeit with some slight positional or quantitative differences. Therefore, distances less than 0.3 implied that the two sets could be considered consistent. In contrast, distances between 0.3 and 0.4, as shown in Figure 13d, indicated differences in the overall layout between expert B and expert C. While the two sets of lines varied in their overall structure, some lines had similar positions. If the distance between two sets exceeded 0.4, as depicted in Figure 13c, the results between the algorithm and the experts exhibited significant differences in both the overall layout and the positions of individual lines. Hence, distances greater than 0.3 signified inconsistency between two sets of lines. Supplementary to the results in Figure 13, other quantitative results on the differences between the model and the experts are appended in Appendix B. 

**Figure 14:**
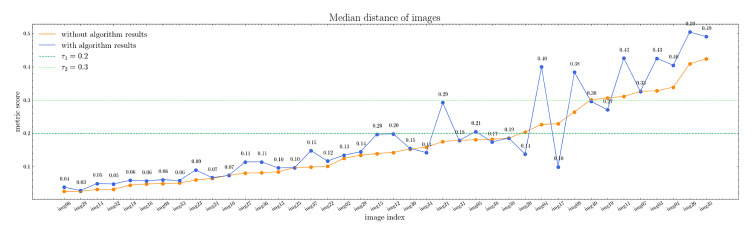
Distance analysis between line sets: The orange line illustrates the median distance of the four experts. The blue line illustrates the median distance of the five experts, where the result of the model is regarded as the fifth expert.

Subsequently, we established two thresholds for these distances: $\tau_{1}=0.2$ was the upper limit distance for two sets indicating overall agreement, while $\tau_{2}=0.3$ served as the upper limit distance for two sets with acceptable differences. To assess the correlation between the algorithm and the four experts, we calculated the median distances both with and without the algorithm’s results. The results are presented in Figure 14, the orange line represents the median distance between the four experts; the blue line signifies the median distance between all five sets, including the algorithm’s set. As indicated in the figure, the algorithm’s results were consistent with experts, except for specific images that exhibited inconsistencies even among the experts.

In our empirical study of this model, we compared it with a random distribution and analyzed the consistency of its results with expert labeling. The experimental findings demonstrated the model’s effectiveness in reconstructing leading lines in images. Although it may exhibit bias in some specific compositions, as seen in Figure 13c, the model proved applicable in the majority of scenarios for reconstructing leading lines, as evident in Figure 12.

## 6. Application

The reconstruction of leading lines aids in comprehending an image composition, making our proposed model a valuable tool for photographers.

**Figure 15:**
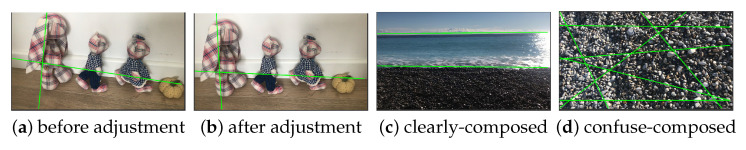
Applications of leading lines. (**b**) is the optimized result of (**a**) with the guide of leading lines displayed with green color. (**c**) Clearly-composed sea beach, (**d**) Pebble beach without clear composition.

We have integrated our model into a camera, enabling the position of leading lines to adapt to the captured content, thus offering photographers a compositional guide. As depicted in Figure 15a represents the initial composition, while Figure 15b showcases the composition adjusted based on the position of leading lines.

Moreover, the position and number of leading lines also contribute to capturing well-composed images. An abundance of disorganized leading lines in a camera shot often indicates an unremarkable image composition, as seen in Figure 15d. In contrast, clearly composed images with distinct themes tend to feature concise and well-organized leading lines, exemplified in Figure 15c. The leading lines reconstruction algorithm provides an objective reference for assessing image composition.

## 7. Conclusions and Future Works

In this paper, we have demonstrated a consensus among experts in the identification of composition leading lines within images, highlighting the potential utility of automatic reconstruction for image composition analysis and assistance during image capture. To detect probable leading lines, we have introduced an unsupervised automatic algorithm, which initially calculates all potential weighted leading lines in an image based on pixel contrast. Subsequently, it groups the weighted leading lines, ultimately generating the final leading lines by identifying the centers of the groups. Acknowledging the subjective variability of leading lines, we have devised a metric for quantifying the distance between two sets of lines, allowing comparisons between sets with varying numbers of lines. In addition to conducting several subjective studies, we have performed objective comparisons to evaluate the accuracy and robustness of our algorithm.

For future work, we aspire to address other forms of leading curves, particularly focusing on circular leading curves. We also aim to expand our ground truth dataset of image composition leading lines to pave the way for supervised algorithms capable of handling more intricate compositions and subjective preferences. 

## Figures and Tables

**Table 1:** Results of the 2AFC Experiment.

Condition	Choices for the Model	Other Choices	Percent
Our method vs. Random	559	41	93.2
Our method vs. Expert	192	408	32.0

**Figure A1:**
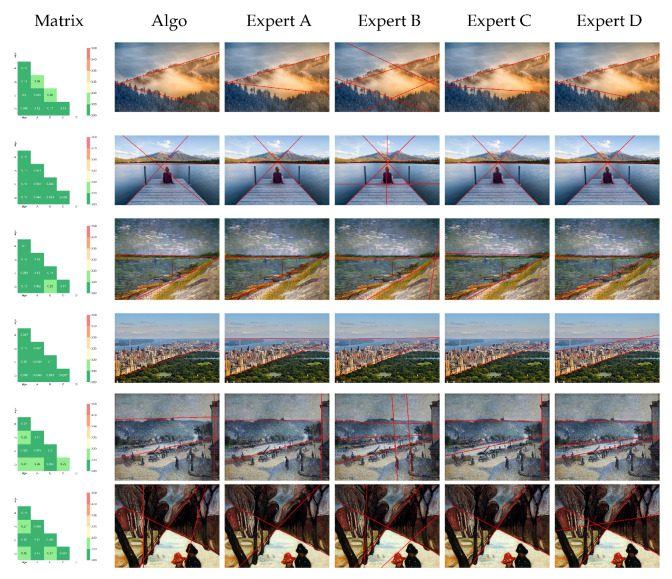
Comparison results and matrices.

## Data Availability

All the data used in this paper are available on these collections: https://kaizhao.net/nkl [16]; https://www.clevelandart.org/art; https://www.kaggle.com/datasets/ikarus777/best-artworks-of-all-time All of the images with credits and leading lines results can be read at: https://projets.jrcandev.netlib.re/leadinglines (accessed on 22 December 2023).

## Appendix A

The distance from the line set F to the line set G is computed as:

(A1) $$d_{LS}\left(F,G\right)=\frac{1}{N_{F}}\sum_{i=1}^{N_{F}}d_{LS}\left(f_{i},G\right)$$

The distance from the line set G to the line set F is computed as:

(A2) $$d_{LS}\left(G,F\right)=\frac{1}{N_{G}}\sum_{i=1}^{N_{G}}d_{LS}\left(g_{i},F\right)$$

where $N_{F}$ is the number of lines in the set F, and $N_{G}$ is the number of lines in the set G. Then we use the average of two distance as the final distance between two sets, which is computed as:

(A3) $$D_{LS}\left(F,G\right)=\frac{1}{2}\left(d_{LS}\left(F,G\right)+d_{LS}\left(G,F\right)\right)$$

## Appendix B

A comprehensive compilation of all comparative results is accessible via the following URL https://projets.jrcandev.netlib.re/leadinglines (accessed on 22 December 2023).

These results seem to support the hypothesis that the model’s output closely aligns with that of the experts. Beyond the quantitative dimension, it is notable that the model’s propositions, even when divergent from the average consensus, do not appear anomalous and occasionally coincide with the judgments of one of the experts. To investigate this proposition, further examinations involving a broader spectrum of experts would be requisite.
